# The Relationship of Waist Circumference with the Morbidity of Cardiovascular Diseases and All-Cause Mortality in Metabolically Healthy Individuals: A Population-Based Cohort Study

**DOI:** 10.31083/j.rcm2506212

**Published:** 2024-06-13

**Authors:** Yue Su, Jinyu Sun, Ying Zhou, Wei Sun

**Affiliations:** ^1^Department of Cardiology, The First Affiliated Hospital of Nanjing Medical University, 210029 Nanjing, Jiangsu, China

**Keywords:** waist circumference, cardiovascular disease, morbidity, all-cause mortality

## Abstract

**Background:**

This study explores the relationship between waist circumference and morbidity of cardiovascular diseases (CVD) and all-cause mortality in metabolically healthy individuals.

**Methods:**

A cohort of 5775 metabolically healthy participants from the 2001–2014 US National Health and Nutrition Examination Survey and National Death Index database was tracked over a median period of 81 months. These participants were divided into quartiles (Q1, Q2, Q3, Q4) based on increasing waist circumference. To compensate for missing covariates, multivariate multiple imputation methods were used. Adjusted logistic regression models were employed to examine the correlation between waist circumference and cardiovascular disease prevalence. Furthermore, Kaplan-Meier curves and multivariable Cox regression analysis were utilized to evaluate the association between waist circumference and all-cause mortality, both qualitatively and quantitatively.

**Results:**

The adjusted logistic regression model indicated that a 10 cm increase in waist circumference was associated with a 1.45 times higher prevalence of CVD. As a categorical variable, there was a significant upward trend in CVD incidence across quartiles of waist circumference. The adjusted odds ratios (95% confidence intervals) were 2.41 (1.13–5.53) for Q2, 2.65 (1.18–6.39) for Q3, and 2.53 (0.9–7.44) for Q4, compared to Q1. Notably, individuals with high waist circumference showed significantly poorer survival compared to those with low waist circumference (*p* = 0.008). The Cox regression analysis revealed that each 10 cm increase in waist circumference contributed to an ~8% increase in all-cause mortality.

**Conclusions:**

This study underscores a positive correlation between waist circumference and both CVD morbidity and all-cause mortality in metabolically healthy individuals. The findings highlight the significance of routinely monitoring waist circumference for effective CVD risk management, regardless of metabolic health status.

## 1. Introduction

Cardiovascular disease (CVD) is a recognized leading global reason for mortality and financial burden, comprising a set of heterogeneous circulatory systems and heart disorders [1]. It is widely acknowledged that the increased morbidity and mortality of CVD are significantly correlated with cardiovascular-associated metabolic diseases, such as hypercholesterolemia, hypertension, and hyperglycemia [2]. Even when a healthy metabolic status is maintained during long periods of time, there are still many metabolically healthy patients suffering from CVD. Moreover, the underlying risk factors and pathogenesis of CVD remain unclear [3].

Previous cohort studies demonstrated that obesity, independent of major metabolic factors, seems to be a significant risk factor for CVD [4]. Subsequently, several cohort studies investigated the association between metabolic health status and CVD risk, with a particular focus on the conversion from metabolically healthy obesity (MHO) to an unhealthy metabolic situation. Many of these studies arrived at a positive conclusion [4, 5]. However, the correlations between MHO and the incidence of CVD or all-cause mortality, remained unclear when compared to metabolically healthy individuals with a normal weight [5, 6, 7, 8, 9, 10]. While meta-analyses [6, 7] and large-scale cohort research [8, 9] have shown a higher risk of cardiovascular events in the MHO group, the findings are not consistently conclusive [5, 10].

Obesity is commonly defined by the body mass index (BMI), a measurement that is insufficient to find out the association between regional body fat distribution and cardiovascular-specific morbidity [11]. As shown in previous studies, abdominal fat was related to the risk of CVD and cardiovascular-related metabolic risk factors [12, 13], which could be simply and precisely estimated by waist circumference [14]. The increasing risk of cardiovascular disorders is related to high body fat in the abdominal region by causing cardio-metabolic stresses, even in normal BMI ranges [15]. However, it remains indistinct whether waist circumference is correlated with all-cause mortality of CVD patients with normal metabolic profiles.

Therefore, we aimed to explore the association of waist circumference with cardiovascular-specific morbidity and all-cause mortality in metabolically healthy individuals.

## 2. Methods

### 2.1 Data Source and Study Population

The National Health and Nutrition Examination Survey (NHANES) is a comprehensive project mainly designed to assess the nutritional and health status of American civilians. It involves in-depth interviews, physical examinations, and laboratory tests. National Death Index (NDI) serves duty as an authoritative database containing death record data in the United States, which assists in tracking the relationship between risk factors and mortality. Seven consecutive NHANES cycles from 2001–2014 and corresponding follow-up information from the NDI database were collected in this study.

We included participants with body measurement parameters, dietary and medical information, risk factors of cardiovascular disease, history and mortality of the cardiovascular disease, and normative biochemistry profile. The exclusion criteria included: (1) participants aged <18 or >80 years, (2) deceased within three months of all causes, (3) diagnosed with cancer, (4) had missing data, (5) pregnant individuals, (6) individuals meeting one of the following conditions were defined as metabolic unhealthy: systolic blood pressure ≥130 mmHg and/or diastolic blood pressure ≥85 mmHg, triglycerides ≥150 mg/dL, high-density lipoprotein cholesterol <1.0 mmol/L (male) or 1.3 mmol/L (female), and fasting plasma glucose ≥100 mg/dL [16]. The National Center for Health Statistics Research Ethics Review Board approved this study and obtained informed consent from all participants.

### 2.2 Waist Circumference

The trained examiner measured the waist circumference directly on the skin at the superior lateral border of the iliac crest using standardized techniques and equipment. Specifically, the examiner stood on the participant’s right side and located the pelvis’s right ilium by palpating the hip area. Then, the examiner marked a horizontal line above the most superior lateral border and extended a steel tape around the waist at this line. Significantly, waist circumference should be determined after exhaling one normal breath. Following measurement, waist circumference would be divided into quartiles, and the lowest quartile (Q1) was identified as the reference group.

### 2.3 CVD Definition and Survival Outcomes

The history of CVD was provided by a standardized self-reported personal interview data named medical conditions section 160b-f (MCQ160b-f). Individuals were diagnosed as CVD if answering “yes” to either of the following questions: “Has a doctor or other health professional ever told you that you have coronary heart disease (CHD)/congestive heart failure (CHF)/heart attack/angina pectoris/stroke?” [17].

Individual cardiovascular-specific mortality status was acquired from the NDI database, defined as death caused by cerebrovascular and heart disease. Cardiovascular-specific mortality includes acute rheumatic fever and chronic rheumatic heart disease (I00–I09), hypertensive heart disease (I11), hypertensive heart and renal disease (I13), ischemic heart disease (I20–I25), other heart disease (I26–I51), or cerebrovascular disease (I60–I69).

### 2.4 Covariates

This study encompassed a diverse range of participants, including various demographics, cardiometabolic risk factors, and behavioral risk factors. Demographic variables were obtained from standard questionnaires, including age, gender, ethnicity and income level. The race was composed of Mexican American, non-Hispanic white, non-Hispanic black, other Hispanic, and individuals from other ethnic backgrounds. Income level was assessed using the family income-to-poverty ratio (PIR), which is calculated by dividing family income by the federal poverty level. The PIR categories were defined as <1.33, 1.33–3.50, and ≥3.50 [18]. Cardiometabolic risk factors consisted of body measurements, lipid profiles, blood glucose, blood pressure, chronic kidney disease, sodium intake, smoking status, alcohol consumption, and physical activity [19, 20]. The estimated glomerular filtration rate (eGFR) was determined using the Chronic Kidney Disease-Epidemiology Collaboration equation [21]. Smoking and drinking were recognized as consuming more than 100 cigarettes in life and 12 alcoholic drinks per year, respectively. Insufficient total leisure-time physical activity was defined as engaging in less than 150 minutes of moderate- or vigorous-intensity activity per week [22]. BMI calculation involved dividing an individual’s weight in kilograms by the square of their height in meters. The procedures and protocols for the questionnaires and physical and laboratory examinations were detailed on the NHANES website.

### 2.5 Statistical Analysis

We initially employed the Kolmogorov-Smirnov test to assess normality and utilized multivariate multiple imputation methods with five replications to fill in missing covariates, maximizing statistical power [23, 24]. Normally distributed continuous variables were presented as mean ± standard deviation, while abnormally distributed variables were expressed as median with inter-quartile range. Categorical variables were expressed as percentages.

We compared the baseline characteristics of cardiovascular-related covariates across different waist circumference levels by respectively applying the one-way analysis of variance (ANOVA) test for normally distributed variables, Kruskal-Wallis test for non-normally distributed variables, or chi-square test for categorical variables. Subsequently, we applied three different logistic regression models to determine the odds ratios (ORs) with corresponding 95% confidence intervals (95% CIs) regarding the association between waist circumference and the prevalence of cardiovascular: (1) unadjusted model; (2) minimally-adjusted model accounting for sex, age, race, PIR, education, triglycerides, total-to-high density lipoprotein (HDL) cholesterol, smoking, and drinking; (3) fully-adjusted model additionally considering BMI. Moreover, a restricted cubic spline was used to illustrate the relationship between waist circumference and cardiovascular disorder prevalence by employing five knots located at specific percentiles (5th percentile, 27.5th percentile, 50th percentile, 72.5th percentile, and 95th percentile), with median waist circumference serving as the reference point [25]. Following this analysis, multivariable Cox regression analysis was conducted to evaluate the correlation between waist circumference and all-cause mortality and calculated the non-adjusted and adjusted hazard ratio (HR) with 95% CIs. Bonferroni-Holm method was applied to adjust the survival comparison among groups. Finally, survival outcomes were shown by Kaplan-Meier curves across waist circumference levels and compared with the log-rank test [26, 27].

All statistical analyses were performed by R software (version 4.1.1; R Foundation for Statistical Computing, Vienna, Austria). *p* < 0.05 was identified as having statistical significance.

## 3. Results

### 3.1 Characteristics of the Study Population

A total of 5775 participants were enrolled in this study, and their baseline characteristics are shown in Table 1. The prevalence of CVD was 1.9%, with the waist circumference ranging from 55.5 to 157.0 cm and a median (Q1, Q3) of 87.1 (79.2, 96.0) cm. Over a median follow-up of 81 months, 143 (2.5%) all-cause mortalities were observed. The median age of the participants was 35 years, and 44.3% were males. Participants with higher waist circumference levels were more likely to be older, male, obese, and smoking. When compared to those with lower waist circumference, their metabolic symbolism seemed to be more related to the occurrence of cardiovascular diseases, such as higher levels of blood glucose, blood pressure and lipid profile. Furthermore, as waist circumference increased, both morbidity and mortality rates rose. However, there were no significant differences in CVD mortality, alcohol consumption, PIR level, or sodium intake.

**Table 1.** **Demographic characteristics of the participants**.

Variables		Overall	Groups divided by waist circumference				*p*-value
			Q1 [55.5, 79.2]	Q2 [79.2, 87.1]	Q3 [87.1, 96.0]	Q4 [96.0, 157.0]	
Number of participants		5775	1445	1449	1461	1420	
Age (years), (median [Q1, Q3])		35.0 [26.0, 46.0]	29.0 [23.0, 40.0]	34.0 [25.0, 43.0]	37.0 [29.0, 48.0]	39.0 [29.0, 51.0]	<0.001
Gender (Male), *n* (%)		2557 (44.3)	446 (30.9)	595 (41.1)	734 (50.2)	782 (55.1)	<0.001
Race, *n* (%)							<0.001
	Non-Hispanic White	2680 (46.4)	647 (44.8)	684 (47.2)	682 (46.7)	667 (47.0)	
	Non-Hispanic Black	1161 (20.1)	292 (20.2)	242 (16.7)	278 (19.0)	349 (24.6)	
	Mexican American	926 (16.0)	156 (10.8)	224 (15.5)	285 (19.5)	261 (18.4)	
	Other Hispanic	467 (8.1)	101 (7.0)	140 (9.7)	129 (8.8)	97 (6.8)	
	Other races	541 (9.4)	249 (17.2)	159 (11.0)	87 (6.0)	46 (3.2)	
PIR level, *n* (%)							0.58
	<1.33	1513 (26.2)	402 (27.8)	378 (26.1)	364 (24.9)	369 (26.0)	
	1.33–3.5	1877 (32.5)	466 (32.2)	472 (32.6)	467 (32.0)	472 (33.2)	
	≥3.5	2385 (41.3)	577 (39.9)	599 (41.3)	630 (43.1)	579 (40.8)	
Education, *n *(%)							0.001
	Below high school	1075 (18.6)	232 (16.1)	256 (17.7)	292 (20.0)	295 (20.8)	
	High School	1186 (20.5)	293 (20.3)	288 (19.9)	282 (19.3)	323 (22.7)	
	Above high school	3514 (60.8)	920 (63.7)	905 (62.5)	887 (60.7)	802 (56.5)	
Waist circumference (cm), (median [Q1, Q3])		87.1 [79.2, 96.0]	74.6 [71.6, 77.2]	83.2 [81.2, 85.3]	91.3 [89.2, 93.4]	103.6 [99.2, 110.3]	<0.001
BMI (kg/$m^{2}$), (median [Q1, Q3])		24.8 [22.1, 28.2]	20.7 [19.3, 22.0]	23.5 [22.3, 24.9]	26.1 [24.6, 27.8]	30.8 [28.4, 34.2]	<0.001
WCBMI, (median [Q1, Q3])		3.5 [3.3, 3.7]	3.6 [3.4, 3.8]	3.5 [3.4, 3.7]	3.5 [3.3, 3.7]	3.4 [3.2, 3.6]	<0.001
Cardiovascular diseases, *n* (%)		111 (1.9)	10 (0.7)	26 (1.8)	36 (2.5)	39 (2.7)	<0.001
All-cause mortality, *n *(%)		143 (2.5)	27 (1.9)	33 (2.3)	31 (2.1)	52 (3.7)	0.009
Follow-up time (months), (median [Q1, Q3])		81.0 [47.0, 126.0]	82.0 [47.0, 127.0]	81.0 [46.0, 122.0]	82.0 [50.0, 129.0]	81.0 [46.0, 125.0]	0.162
Blood pressure							
	SBP (mmHg), (median [Q1, Q3])	111.3 [104.7, 117.3]	108.0 [101.3, 114.7]	110.0 [104.0, 116.7]	112.0 [105.3, 118.7]	114.0 [107.3, 120.0]	<0.001
	DBP (mmHg), (median [Q1, Q3])	67.3 [61.3, 72.7]	66.0 [60.0, 71.3]	66.7 [60.7, 72.0]	67.3 [62.0, 72.7]	68.7 [62.7, 74.0]	<0.001
Lipid profile							
	Total-to-HDL cholesterol ratio, (median [Q1, Q3])	3.1 [2.6, 3.6]	2.7 [2.4, 3.1]	2.9 [2.5, 3.4]	3.2 [2.7, 3.8]	3.4 [2.9, 4.1]	<0.001
	Triglyceride level (mg/dL), (median [Q1, Q3])	78.0 [58.0, 104.0]	67.0 [51.0, 88.0]	75.0 [56.0, 100.0]	83.0 [62.0, 108.0]	91.0 [68.8, 115.0]	<0.001
Blood glucose							
	HbA1c (%), (median [Q1, Q3])	5.3 [5.0, 5.5]	5.2 [5.0, 5.4]	5.2 [5.0, 5.4]	5.3 [5.1, 5.5]	5.4 [5.1, 5.5]	<0.001
	FPG (mg/dL), (median [Q1, Q3])	86.0 [81.0, 91.0]	84.0 [79.0, 88.0]	85.0 [80.0, 90.0]	87.0 [82.0, 92.0]	89.0 [84.0, 93.0]	<0.001
eGFR (mL/min/1.73 $m^{2}$), (median [Q1, Q3])		114.1 [93.8, 139.6]	98.7 [82.5, 113.9]	109.1 [92.3, 127.8]	119.9 [100.3, 139.6]	141.9 [113.1, 173.9]	<0.001
Health behavior							
	Smoking status, *n *(%)	2215 (38.4)	502 (34.7)	557 (38.4)	560 (38.3)	596 (42.0)	0.001
	Alcohol consumption, *n *(%)	623 (10.8)	161 (11.1)	143 (9.9)	153 (10.5)	166 (11.7)	0.422
	Sufficient physical activity, *n* (%)	1342 (23.2)	299 (20.7)	362 (25.0)	340 (23.3)	341 (24.0)	0.042
	Sodium intake (mg), (median [Q1, Q3])	3209.0 [2309.0, 4521.5]	3189.0 [2255.0, 4526.0]	3178.0 [2296.0, 4433.0]	3188.0 [2309.0, 4461.0]	3316.0 [2387.2, 4681.2]	0.266

Abbreviations: PIR, poverty-income ratio; BMI, body mass index; WCBMI, body mass index-adjusted waist circumference; SBP, systolic blood pressure; DBP, diastolic blood pressure; HDL, high density lipoprotein; HbA1c, hemoglobin A1c; FPG, fasting plasma glucose; eGFR, estimated glomerular filtration rate.

### 3.2 The Association between Waist Circumference and the Prevalence of CVD

Table 2 summarizes the relationship between waist circumference and the prevalence of CVD. In the fully adjusted logistic model, each 10 cm increase in waist circumference led to a 1.45 times higher prevalence of CVD. As a categorical variable, there was a significant upward trend in CVD incidence across quartiles of waist circumference in the fully adjusted model. The adjusted odds ratios (95% confidence intervals) were 2.41 (1.13–5.53) for Q2, 2.65 (1.18–6.39) for Q3, and 2.53 (0.9–7.44) for Q4, compared to Q1. The restricted cubic splines visualized a nonlinear relationship between waist circumference and prevalence of CVD when set the median waist circumference as reference (**Supplementary Fig. 1**). An elevated prevalence of CVD was observed with the increase in waist circumference around the reference.

**Table 2.** **The association of waist circumference with cardiovascular diseases prevalence using logistic regression models**.

		Non-adjusted model		Minimally-adjusted model		Fully-adjusted model	
		Odds ratio	*p*-value	Odds ratio	*p*-value	Odds ratio	*p*-value
Waist circumference (Per 10 cm)		1.31 (1.15–1.48)	<0.001	1.1 (0.94–1.28)	0.23	1.45 (0.99–2.13)	0.055
Categories							
	Q1 [55.5, 79.2]	Reference		Reference		Reference	
	Q2 [79.2, 87.1]	2.62 (1.30–5.73)	0.01	2.25 (1.08–5.01)	0.036	2.41 (1.13–5.53)	0.029
	Q3 [87.1, 96.0]	3.63 (1.86–7.74)	<0.001	2.33 (1.15–5.14)	0.025	2.65 (1.18–6.39)	0.023
	Q4 [96.0, 157.0]	4.05 (2.10–8.61)	<0.001	2 (0.97–4.45)	0.071	2.53 (0.9–7.44)	0.083

Minimally-adjusted model: We adjusted for sex, age, race, PIR, education, triglycerides, total-to-HDL cholesterol, smoking, and drinking. Fully-adjusted model: We adjusted for sex, age, race, PIR, education, triglycerides, total-to-HDL cholesterol, smoking, drinking, and BMI. Abbreviations: PIR, poverty-income ratio; HDL, high density lipoprotein; BMI, body mass index.

### 3.3 The Association between Waist Circumference and All-Cause Mortality

**Fig. 1.**
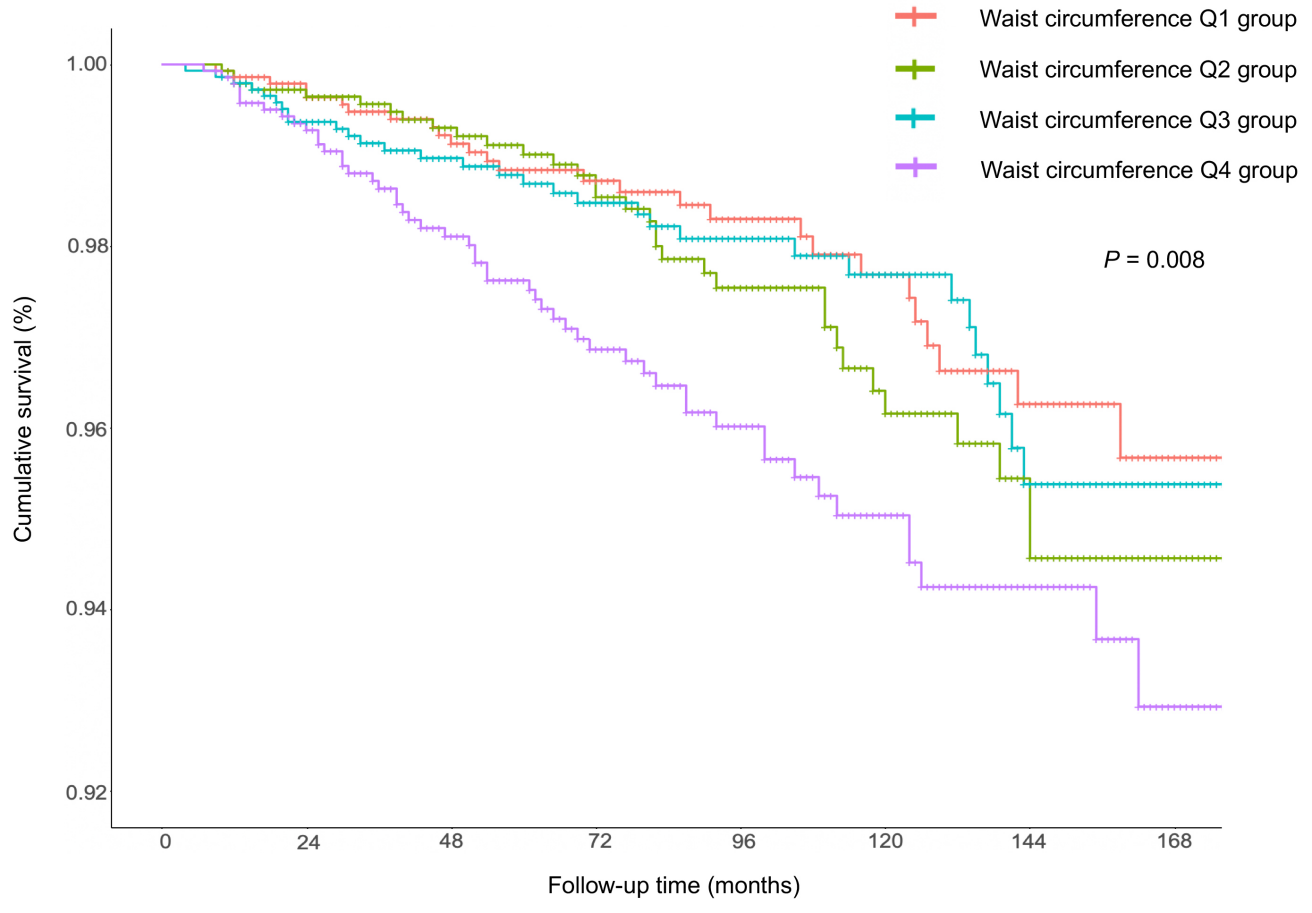
**Kaplan-Meier plots in metabolically healthy population by quartiles of waist circumference.** The Bonferroni-Holm method was utilized to account for the survival comparison among four groups. Compared to the Q1 group, *p* value in the Q2, Q3, and Q4 group are 0.524, 0.663 and 0.018, respectively. Q1: 55.5–79.2 cm; Q2: 79.2–87.1 cm; Q3: 87.1–96.0 cm; Q4: 96.0–157.0 cm.

As shown in Kaplan-Meier curve analyses (Fig. 1), participants in the high waist circumference group showed significantly poorer survival compared with the low waist circumference group (*p* = 0.008). Multivariable Cox regression analysis (Table 3) revealed that each 10 cm increase in waist circumference would contribute to an ~8% increase in all-cause mortality. Notably, waist circumference exhibited a significant and positive association with all-cause mortality in the fully-adjusted model (HR, 1.08; 95% CI, 1.02–1.13), whereas the statistical significance disappeared in the non-adjusted model (HR, 1.00; 95% CI, 0.98–1.02) and minimally-adjusted model (HR, 1.02; 95% CI, 1.00–1.04).

**Table 3.** **The association of waist circumference with all-cause mortality using Cox regression model**.

	Non-adjusted model		Minimally-adjusted model		Fully-adjusted model	
	Hazard Ratio	*p*-value	Hazard Ratio	*p*-value	Hazard Ratio	*p*-value
Waist circumference (Per 10 cm)	1.00 (0.98, 1.02)	0.91	1.02 (1.00, 1.04)	0.052	1.08 (1.02, 1.13)	0.004

Minimally-adjusted model: We adjusted for sex, age, race, PIR, education, triglycerides, total-to-HDL cholesterol, smoking, and drinking. Fully-adjusted model: We adjusted for sex, age, race, PIR, education, triglycerides, total-to-HDL cholesterol, smoking, drinking, and BMI. Abbreviations: PIR, poverty-income ratio; HDL, high density lipoprotein; BMI, body mass index.

## 4. Discussion

Obesity is recognized as a heterogeneous disease, with the distribution of fat in various body regions contributing to differing cardiovascular and metabolic risks. BMI provides a general measure of obesity but fails to account for the distribution of fat and muscle mass. This limitation un-dermines BMI’s effectiveness in assessing risks associated with obesity-related diseases. In contrast, waist circumference is a readily accessible anthropometric index that evaluates abdominal fat distribution. Notably, a high waist circumference is an independent risk factor for a variety of cardiovascular diseases and all-cause mortality, irrespective of BMI [28, 29, 30, 31]. In light of these findings, the expert consensus from the International Atherosclerosis Society and the International Society for Cardiometabolic Risk strongly recommends routine measurement of waist circumference as a critical ‘vital sign’ for a comprehensive assessment of metabolic risks associated with fat distribution [32].

Our study highlights the following key findings: (1) The higher waist circumference was associated with the increasing prevalence of CVD in metabolic healthy individuals; (2) A positive relationship was observed between waist circumference and all-cause mortality in those without metabolic disorders; (3) Measuring waist circumference in metabolically healthy populations holds potential as a valuable tool for CVD prevention.

Our previous research showed that waist circumference is independently and positively correlated with all-cause mortality in individuals with hypertension (HR, 1.44; 95% CI, 1.33–1.57). In contrast, this study specifically addressed individuals without metabolic abnormalities, and a 1.45 times higher CVD prevalence was observed for every 10 cm increase in waist circumference within the fully adjusted model. This finding was consistent when waist circumference was treated as a categorical variable, with the CVD morbidity of the Q4 group being 2.53-fold compared to the Q1 group. Furthermore, the positive relationship between waist circumference and all-cause mortality persisted after multivariable Cox regression analysis, indicating that the adverse effects of a high waist circumference may not be solely attributed to cardiometabolic abnormality. While previous research has indicated a U-shaped relationship between waist circumference and CVD incidence, our research employed a restricted cubic spline and revealed a nonlinear curve. This difference may be attributed to the distinct population we examined. Our study focused on younger individuals who were metabolically healthy, while former studies were based on the general population with various metabolic disorders. In subjects with ischemic heart disease, a phenomenon known as the “obesity paradox” has been observed, where higher waist circumference or BMI is linked to improved prognosis [33]. Despite obesity being associated with increased risk factors for established CVD, it has been observed that overweight or obese individuals may have a more favorable outcome compared to those who are leaner in many types of CVD [34]. Potential reasons for the obesity paradox in CVD include: (1) greater metabolic reserves, (2) non-purposeful weight loss, (3) protective cytokines, (4) less cachexia, (5) young age at presentation, (6) earlier medical intervention, (7) higher blood pressure bring about more cardiac medications, (8) attenuated response to renin–angiotensin–aldosterone system, (9) implications related to cardiorespiratory fitness, (10) increase muscle mass and muscular strength [35]. Crewe C *et al*. [36] found that when adipocytes experience mitochondrial stress, they release small extracellular vesicles into the bloodstream which are then taken up by cardiomyocytes. This leads to compensatory antioxidant signaling in the heart, providing protection against acute oxidative stress and aligning with a preconditioning paradigm [36]. Additionally, other factors such as smoking or unknown confounding variables could potentially mask the true effects of being overweight or obese [37].

To the best of our knowledge, this is the first research to focus on the relationship between waist circumference and all-cause mortality in individuals without metabolic syndrome. Existing research has not provided a consistent conclusion, as previous studies mainly examined whether metabolically healthy but obese individuals face a higher risk of CVD in comparison to those with a normal BMI. A meta-analysis composing 22 prospective studies revealed that participants with MHO had a higher risk of cardiovascular events in comparison to healthy normal-weight individuals, with a pooled risk ratio and confidence interval of 1.45 (1.20–1.70) [6]. This conclusion was supported by another meta-analysis, which additionally suggested that there was no “healthy” pattern of obesity [7]. Large-scale prospective research also indicated a higher risk of long-term cardiovascular events (including cerebrovascular disease, heart failure, coronary heart disease and peripheral vascular disease) in overweight individuals, with heart failure risk nearly doubling after an average 5.4 years of follow-up time [8, 9]. Our results align with previous studies, indicating that higher levels of body fat are correlated with an added risk of CVD and adverse outcomes. Notably, we focused on abdominal fat, which is a compelling marker of visceral adipose tissue. This focus may be due to the fact that individuals who are metabolically healthy but overweight are more likely to develop metabolic complications after a 16-year follow-up [38]. However, studies have shown that the risk of CVD did not increase in individuals maintaining the MHO phenotype defined by BMI [5, 10]. This led us to investigate whether another index could consistently and accurately indicate future CVD risk. Ofstad AP *et al*. [39] explored the relationship between traditional and non-traditional adiposity indices and cardiovascular mortality, and found that sex-specific total body fat index had a stronger association with cardiovascular death than other indices of adiposity. Additionally, we conducted an analysis of the body mass index-adjusted waist circumference (WCBMI) ratio, detailed in **Supplementary Tables 1,2**. The adjusted logistic regression model indicated that per unit increase in WCBMI ratio was associated with a 1.62 times higher prevalence of CVD (*p* = 0.158). The Cox regression analysis revealed that per unit increase in WCBMI ratio contributed to about a 9% increase in all-cause mortality (*p* = 0.068).

The relevance between body fat redistribution and CVD risk has been highlighted by recent studies [40]. Emerging evidence showed that increased gluteofemoral and leg fat mass can be protective factors against cardiometabolic diseases [41, 42, 43], and decrease abdominal adiposity. Abdominal adiposity is easily assessed by waist circumference, primarily indicating excessive visceral fat [44]. This type of abdominal adipose tissue consists mainly of brown adipose tissue, which plays a vital role in the development of abdominal obesity by secreting proinflammatory cytokines and various cytokine-related proteins [45]. Herein, inflammation appears to be the missing link between abdominal obesity and cardiovascular disease [46, 47]. Individuals with abdominal obesity often exhibit high levels of proinflammatory cytokines (tumor necrosis factor-α (TNF-α), interleukin-1α (IL-1α), interleukin-1β (IL-1β), interleukin-6 (IL-6) and interleukin-8 (IL-8)), which can be considered significant prognostic indicators of CVD risk [47, 48]. However, other typical immune-related proteins, such as leptin, macrophage migration inhibitory factor and monocyte chemoattractant protein-1, have not been thoroughly researched in this field [49, 50, 51]. Future studies may explore novel treatments to reduce CVD risk by modulating the influence of these proinflammatory cytokines in metabolically healthy individuals with high waist circumference.

The following limitations should not be ignored: (1) The safety range of waist circumference remains undetermined in this research. Thus, further studies are necessary to provide conclusive evidence in support of our findings. (2) Although we adopted one of the most authoritative definitions of metabolic health according to Lancet Diabetes Endocrinol, additional research is needed to establish a consensus in this regard. (3) This study is cross-sectional and based on the representative U.S. database. As such, it remains uncertain whether our conclusion can be directly extrapolated to different ethnicities and races, accounting for potential variations in lifestyle and genetics. (4) We utilized blood pressure, triglycerides, high-density lipoprotein cholesterol, and fasting plasma glucose as representative profiles for cardiometabolic health based on metabolic syndrome criteria. However, it should be noted that there are additional components such as serum uric acid which play a role in cardiometabolic profiles. Specifically, having hyperuricemia above 5.5 mg/dL has been linked to heightened cardiovascular mortality risk [52]. Hyperuricemia often coexists with being overweight and obesity and may act synergistically with metabolic syndrome components to exacerbate organ damage [52, 53]. The biochemical pathways involved in uric acid production are implicated in the progressive impairment of tissue insulin sensitivity, culminating in adverse lipid profiles, elevated blood pressure, and metabolic disturbances. In future research endeavors, it will be imperative to apply more stringent criteria when defining metabolically healthy profiles to enhance the accuracy and reliability of our findings. (5) Our findings demonstrate a positive relationship between waist circumference and the prevalence of CVD and all-cause mortality. However, it remains unclear how waist circumference impacts the long-term risk of CVD among metabolically healthy individuals. Further investigation is needed in this regard.

## 5. Conclusions

Our research underscores a positive correlation between waist circumference and both CVD morbidity and all-cause mortality in metabolically healthy individuals. The findings highlight the significance of routinely monitoring waist circumference for effective CVD risk management, regardless of metabolic health status.

## Data Availability

The National Health and Nutrition Examination Survey (NHANES) is an open-access population survey dataset. All the data used in this study was acquired from the NHANES.

## Supplementary Material

Supplementary material associated with this article can be found, in the online version, at https://doi.org/10.31083/j.rcm2506212.
