# Supplementary material for: The Relationship of Waist Circumference with the Morbidity of Cardiovascular Diseases and All-Cause Mortality in Metabolically Healthy Individuals: A Population-Based Cohort Study
Source: Rev Cardiovasc Med. 2024 Jun 13;25(6):212. doi: 10.31083/j.rcm2506212 (PMC11270058; doi:10.31083/j.rcm2506212)
Supplement: Supplementary file 1 [file 2153-8174-25-6-212-s1.zip › Supplementary Fig. 1.pdf]

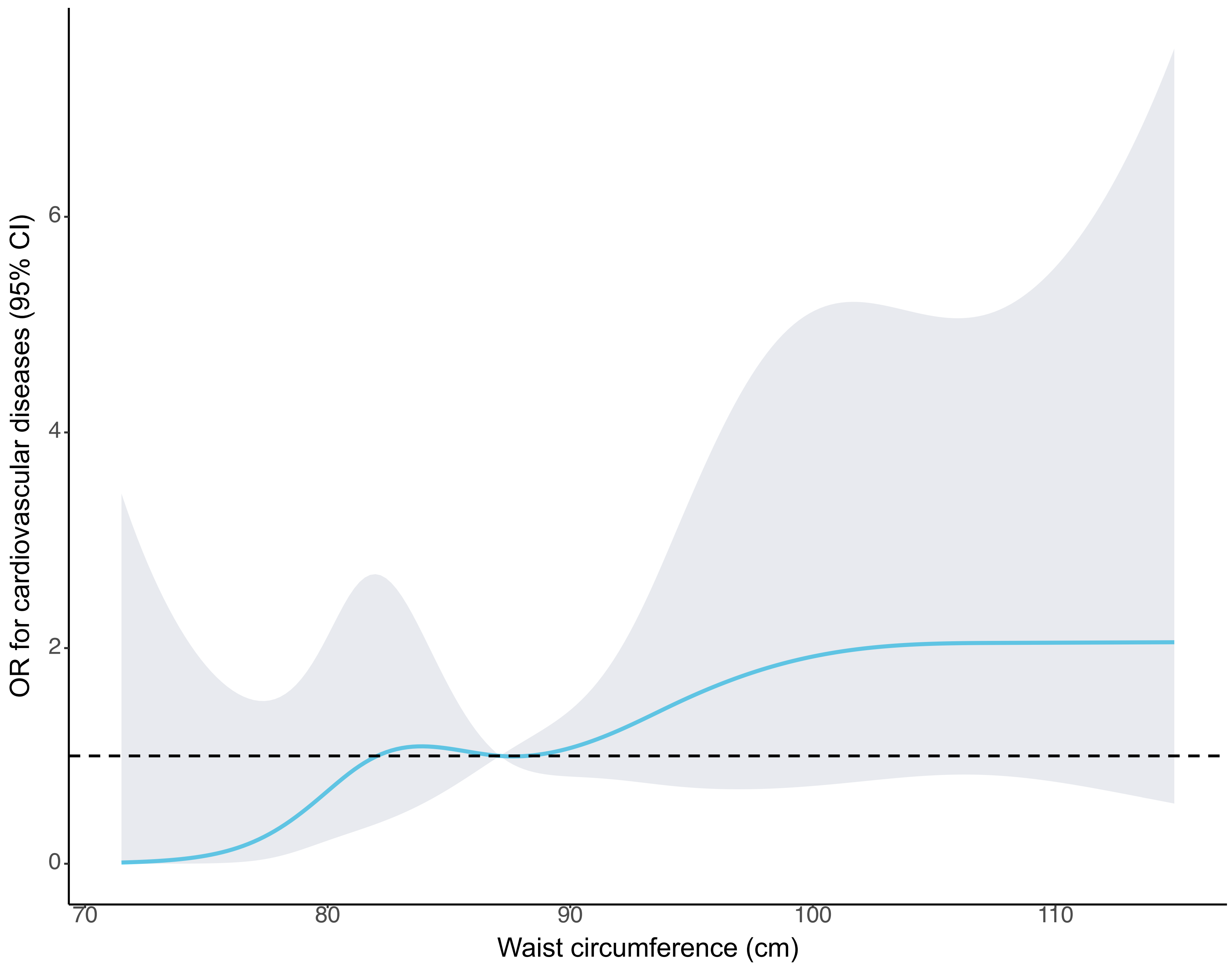

OR for cardiovascular diseases (95% CI)
0
2
4
6
70
80
90
100
110
Waist circumference (cm)

- The adjusted restricted cubic spline model on the association between waist circumference and prevalence of cardiovascular diseases in metabolically healthy individuals. The median waist circumference was set as the reference. Abbreviations: OR: Odds ratio; CI: Confidence interval.
