# Supplementary material for: The Relationship of Waist Circumference with the Morbidity of Cardiovascular Diseases and All-Cause Mortality in Metabolically Healthy Individuals: A Population-Based Cohort Study
Source: Rev Cardiovasc Med. 2024 Jun 13;25(6):212. doi: 10.31083/j.rcm2506212 (PMC11270058; doi:10.31083/j.rcm2506212)
Supplement: Supplementary file 1 [file 2153-8174-25-6-212-s1.zip › Supplementary Tables.pdf]

Supplementary Table 1. The association of WCBMI with cardiovascular diseases prevalence using logistic regression models

	Non-adjusted model		Adjusted model	
	**Odds ratio**	***P*-value**	**Odds ratio**	***P*-value**
WCBMI (Per unit)	6.54 (3.55-12.04)	< 0.001	1.62 (0.83-3.17)	0.158
Categories				
Q1	Reference		Reference	
Q2	0.83 (0.41-1.66)	0.6	0.6 (0.29-1.24)	0.168
Q3	1.68 (0.94-3.09)	0.084	1.06 (0.57-2.04)	0.849
Q4	2.72 (1.61-4.83)	<0.001	1.01 (0.54-1.94)	0.97

Participants were divided into quartiles (Q1, Q2, Q3, Q4) based on increasing WCBMI. In adjusted model, we adjusted for sex, age, race, education, PIR, total-to-HDL cholesterol, triglycerides, smoking, and drinking. Abbreviations: WCBMI, body mass index-adjusted waist circumference; PIR, poverty-income ratio; HDL, high density lipoprotein.

Supplementary Table 2. The association of WCBMI with all-cause mortality using Cox regression model

	Non-adjusted model		Adjusted model	
	**Hazard Ratio**	***P*-value**	**Hazard Ratio**	***P*-value**
WCBMI (Per unit)	1.04 (0.95, 1.13)	0.39	1.09 (0.99, 1.20)	0.068

In adjusted model, we adjusted for sex, age, race, education, PIR, total-to-HDL cholesterol, triglycerides, smoking, and drinking. Abbreviations: WCBMI, body mass index-adjusted waist circumference; PIR, poverty-income ratio; HDL, high density lipoprotein.
